# Potential impacts of a novel integrated extracorporeal-CPR workflow using an interventional radiology and immediate whole-body computed tomography system in the emergency department

**DOI:** 10.1186/s12872-020-01332-4

**Published:** 2020-01-16

**Authors:** Kei Hayashida, Takahiro Kinoshita, Kazuma Yamakawa, Santiago J. Miyara, Lance B. Becker, Satoshi Fujimi

**Affiliations:** 1 Department of Emergency Medicine, Feinstein Institutes for Medical Research, Northwell Health System, 350 Community Dr, Manhasset, NY 11030 USA; 2 Division of Trauma and Surgical Critical Care, Osaka General Medical Center, 3-1-56 Bandai-Higashi, Sumiyoshi-ku, Osaka, 558-8558 Japan; 3 Elmezzi Graduate School of Molecular Medicine, Manhasset, NY USA

**Keywords:** Out-of-hospital cardiac arrest, Extracorporeal cardiopulmonary resuscitation, Emergency department, HERS, Concurrent treatment

## Abstract

Extracorporeal cardiopulmonary resuscitation (ECPR) can be associated with increased survival and neurologic benefits in selected patients with out-of-hospital cardiac arrest (OHCA). However, there remains insufficient evidence to recommend the routine use of ECPR for patients with OHCA. A novel integrated trauma workflow concept that utilizes a sliding computed tomography (CT) scanner and interventional radiology (IR) system, named a hybrid emergency room system (HERS), allowing emergency therapeutic interventions and CT examination without relocating trauma patients, has recently evolved in Japan. HERS can drastically shorten the ECPR implementation time and more quickly facilitate definitive interventions than the conventional advanced cardiovascular life support workflow. Herein, we discuss our novel workflow concept using HERS on ECPR for patients with OHCA.

Extracorporeal cardiopulmonary resuscitation (ECPR) can restore blood circulation in patients with out-of-hospital cardiac arrest (OHCA), as the cardiopulmonary circuit allows immediate stabilization via effective perfusion and gas exchange. Recently, ECPR devices are becoming smaller and cheaper, providing an opportunity to install this rescue therapy in the emergency department (ED) [1]. The international resuscitation guideline mentioned ECPR as a potential support to be considered in settings where it can be rapidly implemented, and for selected patients with a suspected etiology of cardiac arrest that is potentially reversible during a limited period of mechanical cardiorespiratory support [2].﻿﻿ However, there remains insufficient evidence to recommend the routine use of ECPR for patients with OHCA [2].

In 2011, a new integrated trauma workflow concept, with a sliding computed tomography scanner (CT) and interventional radiology (IR) system that enables CT examination and emergency therapeutic intervention, was implemented in Japan. It contains a carbon-fiber fluoroscopic table and all the necessary equipment for life-saving procedures, including airway management, CT examinations, angioembolization, and emergency surgery, which can be performed on the same table (Fig. 1). This integral structure allows physicians to perform both examinations and rapid safe effective procedures, without relocating the patient and is called a hybrid emergency room system (HERS) [3, 4]. We previously demonstrated that the HERS is associated with a decreased mortality in patients with severe blunt trauma compared to that with conventional trauma management, after accounting for potential confounders (adjusted odds ratio [OR], 0.50; 95% confidence interval [CI], 0.29–0.85; *p* = 0.011) [4]. Patients treated with the HERS had shorter time intervals from ER arrival to CT examination and emergency surgery compared to those for conventional management (median [25th–75th percentiles]: 11 [8–16] min vs. 26 [21–32] min, *p* < 0.0001; 47 [37–57] min vs. 68 [51–85] min, p < 0.0001, respectively) [4]. These beneficial observations contributed to the rapid spread of the HERS concept; as of September 2019, eleven tertiary emergency hospitals in Japan and one trauma center in Korea have installed a HERS. This system could be well adapted to ECPR, providing an expedient initiation of mechanical cardiopulmonary support for patients with non-traumatic OHCA.

**Fig. 1:**
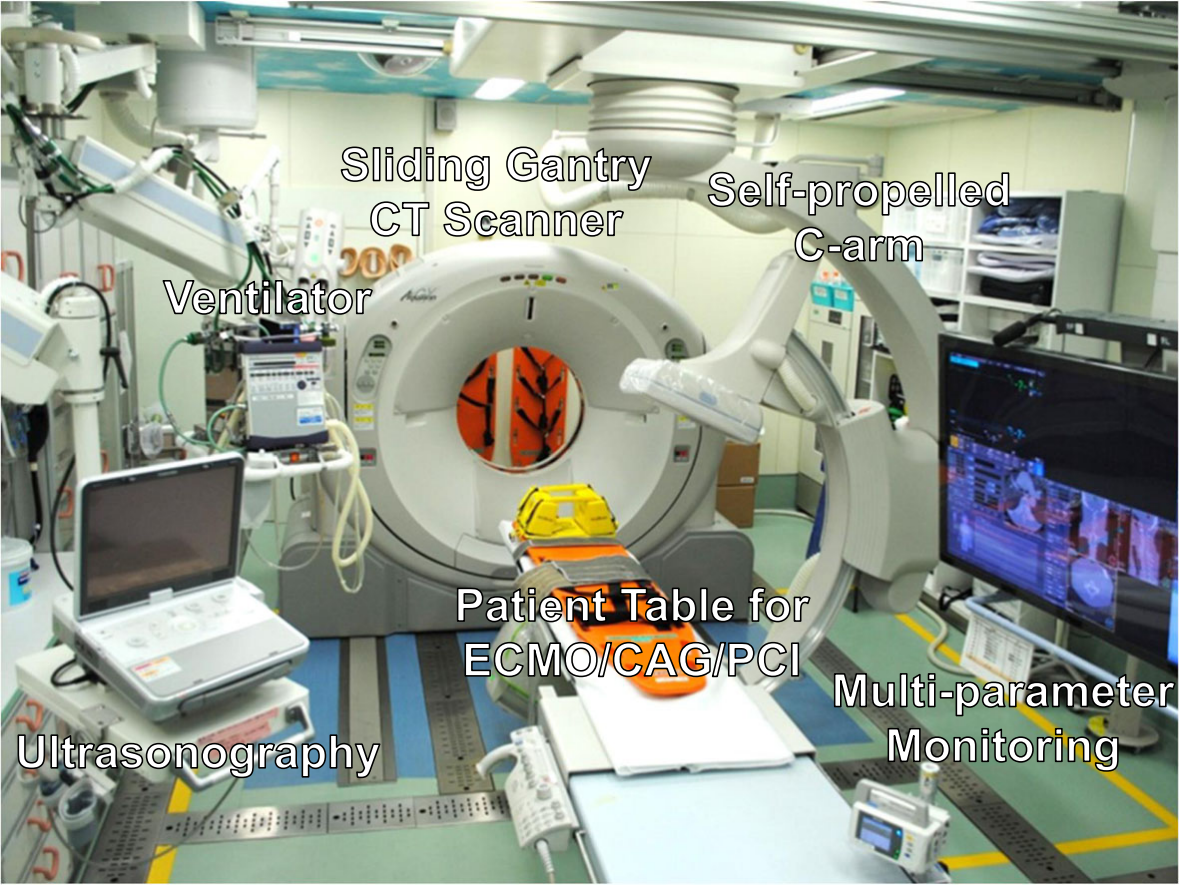
A photograph showing our IR-CT system in the emergency department. All life-saving procedures including airway management, emergency surgery, and transcatheter arterial embolization can be performed on the table without relocating the patient

Immediate high-quality CPR, providing effective oxygenation to the vital organs until ROSC is achieved, is crucial for optimal outcomes. However, there is no consensus regarding the CPR time interval before ECPR implementation. In a single-center retrospective cohort study, the impact of CPR duration on neurological outcomes was evaluated in patients with OHCA; ROSC occurred within 16 min of CPR in 89.7% of patients with good neurological outcomes [5]. In their study, the probability of good functional recovery among all CPR attempts fell to less than 2% when the CPR duration was over 15 min. In another retrospective study including 3992 OHCA patients with resuscitation attempts, refractory OHCA, age, public OHCA, witnessed OHCA, bystander CPR performed, high severity of comorbidity, and resuscitation length > 30 min were all found to be independently associated with increased 30-day mortality. Comparing all patients with a duration of resuscitation > 30 min with duration < 30 min, they found 30-day survival of 38 and 50%, respectively (*P* < 0.001) [6]. In addition, a longer pre-ECPR resuscitation time is significantly associated with a higher mortality rate in patients with OHCA [7, 8]. These currently available data do not suggest that a longer pre-ECPR resuscitation time is a contraindication to ECPR; rather, ECPR should be immediately available to patients who have failed to respond to conventional CPR within the first 30 min. Moreover, as it is reported that the median prehospital resuscitation duration is approximately 30 min [9], ECPR flow should commence for selected patients immediately after hospital arrival, if the patient fails to respond to a short interval of conventional advanced cardiovascular life support (ACLS) in the ED.

Yannopoulos and colleagues [10] evaluated a novel protocol with early transport to a cardiac catheterization laboratory (CCL) for ECPR and revascularization in OHCA patients with refractory ventricular fibrillation/ ventricular tachycardia. In patients who received the early transport protocol, the average time from the 911 call to CCL arrival was 58 ± 17 min, 28/62 (45%) patients survived to hospital discharge, and 26 (42%) had functionally favorable outcomes. In contrast, only 26/170 (15.3%) patients in a historical routine care group survived to hospital discharge with a favorable outcome [OR 4.0, 95%CI 2.08–7.7] [10]. These observations suggest that, in these patients, the early initiation of ECPR to allow for coronary angiography (CAG) and percutaneous coronary intervention (PCI) would likely be more effective than continuing non-invasive conventional ACLS alone.

For treatable etiologies, the objective of ECPR is primarily to increase the chance of ROSC and improve the hemodynamic status, leading to the concept of “treatment bundles” to preserve intact neurological function and improve the long-term prognosis. The treatments for the cause of cardiac arrest should be planned under a time-conscious algorithm. Patients with ROSC and suspected ST-segment elevation myocardial infarction should be considered to receive CAG and possible immediate PCI. If a massive/submassive pulmonary embolism (PE) is likely the etiology, CT pulmonary angiography should be considered. Although the optimal timing of treatment for acute PE remains unclear, due to the absence of randomized controlled trials, subsequent definitive therapies (i.e. thrombolytic therapy, or surgical/catheter embolectomy if thrombolytic therapy is contraindicated or failed) should be performed as soon as possible. If an acute ischemic stroke/intracranial hemorrhage is suspected as a precipitant, head CT should be performed for further interventions. Immediate brain CT after ROSC in patients treated with therapeutic hypothermia can help predict the outcome [11, 12].

The schematic concept of HERS integrated workflow is described in Fig. 2. The HERS enables ACLS to be followed seamlessly by ECPR induction and post-ECPR procedures, including CAG and PCI, without any patient transfer. The HERS likely shortens the time from the 911 call to ECPR initiation and improve survival in OHCA, as early transport contributes to improved outcomes. In addition, the HERS enables safe head and body CT examinations during the post-ECPR phase, even in hemodynamically unstable patients, while in-hospital transport with an extracorporeal membrane oxygenation (ECMO) device implantation can cause considerable harm. For example, when the coronary artery is recognized as intact after ECPR induction followed by CAG, the resuscitation team can seamlessly perform whole-body CT to reveal other treatable etiologies. Vascular complications of ECMO constitute the most important aspect as far as treatment outcomes are concerned [13]. Fluoroscopy with the C-arm allows for immediate and safe cannulations into the femoral artery and vein during ongoing CPR, therefore the HERS might diminish the rates of vascular complications from ECMO placement, including failure to cannulate, accidental cannula displacement, vessel injury, limb ischemia, and retroperitoneal bleeding. Thus, given that an extremely time-conscious algorithm should be adopted in the management of, not only severe trauma but also life-threatening critical illnesses mentioned above, the HERS can provide benefits for cardio-cerebral resuscitation in patients with OHCA or severe medical conditions.

**Fig. 2:**
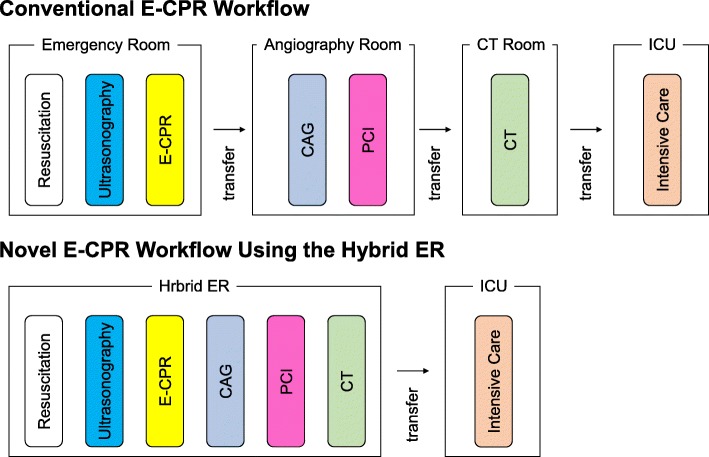
The schematic integrated concept of a hybrid emergency room system (HERS) workflow

Since no/low-flow time intervals are crucial determinants of the outcome, all efforts should be made to minimize the time from cardiac arrest to ECMO flow initiation. Our novel workflow concept using the HERS can drastically shorten the ECPR implementation delay and more quickly facilitate definitive interventions than the conventional ACLS workflow, without relocating the patients. ECPR with the HERS may exponentially accelerate improvements in cardiac arrest management. Further randomized controlled trials with strict inclusion criteria and an appropriate treatment protocol will be required to demonstrate the impact of HERS on ECPR for patients with OHCA.

## Data Availability

The data described in the current manuscript are available from the corresponding author on reasonable request.

## Abbreviations

ACLS: Advanced cardiovascular life support
CAG: Coronary angiography
CCL: Catheterization laboratory
CI: Confidence interval
CPR: Cardiopulmonary resuscitation
CT: Computed tomography
ECMO: Extracorporeal membrane oxygenation
ECPR: Extracorporeal cardiopulmonary resuscitation
ED: Emergency department
HERS: Hybrid emergency room system
IR: Interventional radiology
OHCA: Out-of-hospital cardiac arrest
OR: Odds ratio
PCI: Percutaneous coronary intervention
PE: Pulmonary embolism
ROSC: Return of spontaneous circulation
